# Editorial: New approaches for improving equity in mental health research, treatment, and policy

**DOI:** 10.3389/fpsyt.2025.1666093

**Published:** 2025-09-10

**Authors:** Joanne C. Suarez, Julia Knopes, Lexi C. White, Virginia A. Brown, Edmund Howe

**Affiliations:** ^1^ Prospera Institute, Boston, MA, United States; ^2^ Case Western Reserve University School of Medicine, Cleveland, OH, United States; ^3^ Banner Health, Phoenix, AZ, United States; ^4^ Hastings Center, Garrison, TX, United States; ^5^ Uniformed Services University of the Health Sciences, Bethesda, MD, United States

**Keywords:** psychiatry, mental health, policy, community engaged bioethics, social justice

This Research Topic emerges directly from the shared goals of the *Frontiers in Psychiatry* Editorial Committee and the Frontiers team to advance inclusion, innovation, and equity in mental health research and publishing. As we collectively work to close the gap between scientific discovery and real-world community impact, we recognize that elevating the voices and perspectives of persons living with mental health illnesses and/or conditions is no longer optional—it is essential. This collection of twenty articles collectively endeavors to improve access to essential care for individuals grappling with mental illness. The authors examine the many challenges faced by diverse patient populations who are disadvantaged in their pursuit of mental health support. These more vulnerable individuals differ in age, financial status, race, ethnicity, gender identities, and location—including in both Western and Eastern societies (Hu et al.; Garuma et al.). The articles explore equity in global contexts, demonstrating—through both quantitative and qualitative methods—that mental health is shaped not only by psychotherapeutic or pharmacological treatment, but by structural and social factors like stigma, poverty, cultural beliefs, and family support.

In line with the focus of this Research Topic—*New Approaches for Improving Equity in Mental Health Research, Treatment, and Policy*—we bring forward scholarship that not only engages with underrepresented experiences but also challenges the norms of who participates in, benefits from, and leads mental health research. This vision resonates with broader efforts to push the boundaries of who defines ethical inquiry and to build frameworks that are braver, broader, and more attuned to social justice (1). Such approaches demand that we not only include underrepresented communities but also reshape the ethical foundations of mental health research to reflect their lived realities. Grounded in a commitment to ethical rigor and community relevance, this collection invites authors to help reimagine a research landscape that centers those most affected by psychiatric systems—particularly persons living with conditions that are underacknowledged to others; from underrepresented communities such as Black, Latinx, and LGBTQ+ populations; whose values and experiences are shaped by cultural beliefs beyond biomedicine; and whose access to care is limited by geography or constrained by broader structural inequities.

## Issue overview

Here we provide an overview of selected articles to prime your reading experience.

Let us begin within the French context, where Fond et al. found that people with schizophrenia living in poverty—despite having access to medical care—still experienced worse health outcomes, inconsistent housing, and fewer opportunities for social engagement. Through their study, the authors uncovered new directions for inquiry when noting “surprisingly, individuals in poverty reported higher self-esteem levels compared to their wealthier counterparts”. The results emphasize the importance of examining protective psychological and cultural factors within low-income populations while noting the positive finding that poverty was not associated with diminished social functioning or quality of life. Contributions by Edalati et al. direct attention to the intersection of substance use and mental health concerns, especially within Indigenous and other underserved populations in Canada. The authors highlight that the grip of physiological dependence from substance use may be even harder to overcome than the mental illness itself, and argue for the need to integrate treatment structures across systems to effectively support individuals with co-occurring conditions. Articles in this issue also provide additional context for understanding how mental health conditions intersect with other identities and social locations. For instance, Osman et al. highlight the co-occurrence of anxiety and depression during the postnatal period and challenge the tendency to silo these conditions, underscoring the need for care models that reflect the complexity of lived experience among birthing people whose symptoms may be overlooked or minimized. Contributions by Fu et al. focused on aging populations, specifically those experiencing dementia, while amplifying the critical role community health systems play in supporting recovery and stability for aging populations over time. Here, Camacho et al. present a compelling example of participatory social network research in Colombia by mapping gaps in the mental health service landscape through community collaboration. An approach that mirrors broader calls to integrate structural and intersectional competencies into care delivery Howe (2) such as encouraging providers to ask not only about symptoms but also about external stressors—like whether a patient has transportation to the clinic or access to childcare.

Though many articles in this issue underscore the importance of participatory approaches to mental health research that center the voices of people with lived experiences of mental health conditions, future scholarship should remain mindful of the complexity and nuance of involving psychiatric service users with lived experience of mental health conditions as research collaborators. Scholars like Faulkner (3) and Rose (4) caution that while people with mental health conditions should be engaged as equal partners in research who contribute expertise in the form of lived experience, their voices are often devalued when they do not align with prevailing psychiatric or social scientific understandings of mental health and established methods of collecting and interpreting data. Guidry-Grimes (5) similarly notes that clinicians often fail to engage people with mental health diagnoses in shared decision making, which might extend to a belief in psychiatric and mental health research that people with mental health conditions lack insight and the ability to contribute to or innovate on research. Across several pieces, the literature calls us to carefully assess justice in mental health—both within and beyond clinical settings (6).

Beyond the listed intersectional perspectives, the issue also surfaces urgent ethical dilemmas, such as those surrounding involuntary hospitalization. Aguirre et al. and Howe (7) explore how patients with severe suicidal ideation may resist hospitalization, while providers, fearing liability, may default to hospitalization even when other forms of support are viable. The authors argue that although hospitalization can offer short-term safety, it can also increase long-term trauma and risk. Howe proposes that providers consider strategies rooted in patient autonomy—such as scheduled check-ins and affirming patient self-knowledge—as alternatives to premature hospitalization. These approaches encourage patients to signal when they feel safe versus when additional support is needed, helping to build trust and preserve dignity in the treatment process. As this issue includes calls for affirmative, anti-ableist models of care and scholarship, Yu (8) reminds us that ableism continues to shape assumptions about capacity and worth, especially for people with disabilities and psychiatric diagnoses. Heinze et al. explores mental health outcomes among visually impaired populations, revealing that approximately nine out of ten Black participants expressed feelings of determination and resilience in the face of their visual impairment. The study highlights that black participants, despite systemic challenges encountered with visual limitations, report higher levels of optimism and self-acceptance compared to other groups. While the basis for this optimism remains unclear, the study suggests that cultural and community-based factors may play a protective role—another area ripe for future exploration. With gender and sexual identity emerging as powerful dimensions of analysis, Hambruch et al. and Alibudbud address mental health among LGBTQ+ individuals. They advocate for affirmative psychotherapy practices that validate identity, reduce internalized stigma, and acknowledge the compounding effects of minority stress. As the historical exclusion of women from research is now broadly accepted, and increasing attention is being paid to the mental health needs of birthing people, a call for inclusive language—like “pregnant persons”—is also emphasized as a deliberate act of recognition for individuals who may not identify within traditional gender categories.

Despite the serious challenges highlighted across the Research Topic, many of the authors propose tangible, hopeful solutions. Lei et al. argues that burnout in academic and professional spaces can be mitigated through thoughtful hiring practices and attention to role fit. Ding et al. expands cancer treatment models to consider the emotional needs of patients’ partners, offering a dyadic coping framework that could improve medical outcomes through relational care. These ideas reflect a broader paradigm shift: one that embraces holistic, interconnected understandings of health. Authors also highlight the importance of training and systems-level adaptation. In Turkey, primary care providers with in-service psychiatric training were significantly more confident and effective in prescribing psychotropic medications. As antidepressants, mood stabilizers, and benzodiazepines become more commonly used, this training is increasingly vital. Articles recommend expanded telepsychiatry programs and cross-disciplinary collaborations with pharmacies to increase access and medication literacy, particularly in rural and underserved areas. Elsewhere, innovations include low-tech solutions such as disseminating mental health information via radios during emergencies or leveraging smartphones to expand treatment access in resource-limited settings. Collectively, these strategies demonstrate that impactful change doesn’t always require high-tech interventions—just thoughtful, human-centered design.

## Conclusion

The overall ethical thrust of this Research Topic is to prioritize those who have historically been excluded or underserved. From the structurally marginalized to those navigating conditions, this Research Topic calls for a research and treatment paradigm grounded in collaboration, dignity, and justice. Authors remind us that in order to improve equity in mental health, we must reflect on what kinds of knowledge are valued, whose voices are invited in, and how we design systems that meet people where they are. This Research Topic ultimately asks mental health professionals, researchers, and policymakers to reimagine what’s possible when those most affected by psychiatric systems are seen not just as subjects of research, but as co-creators of solutions. We hope the work shared here sparks new insights and shared commitment to advancing a more just, inclusive, and healing mental health future.

As you read through this Research Topic, we invite you to consider the variety of voices, practice perspectives, and themes that emerge—particularly as this Research Topic highlights how intersecting factors render individuals with serious mental illnesses more vulnerable.
